# Selective Depletion of Antigen-Specific Antibodies for the Treatment of Demyelinating Disease

**DOI:** 10.1016/j.ymthe.2020.11.017

**Published:** 2020-11-17

**Authors:** Wei Sun, Priyanka Khare, Xiaoli Wang, Dilip K. Challa, Benjamin M. Greenberg, Raimund J. Ober, E. Sally Ward

**Affiliations:** 1 Department of Molecular and Cellular Medicine, Texas A&M University Health Science Center, 469 Joe H. Reynolds Medical Sciences Building, 1114 TAMU, College Station, TX 77843, USA; 2 Department of Neurology and Neurotherapeutics, University of Texas Southwestern Medical Center, 5323 Harry Hines Boulevard, Dallas, TX 75390, USA; 3 Department of Biomedical Engineering, Texas A&M University, 5045 Emerging Technologies Building, 3120 TAMU, College Station, TX 77843, USA; 4 Cancer Sciences Unit, Centre for Cancer Immunology, Faculty of Medicine, University of Southampton, Southampton SO16 6YD, UK; 5 Department of Microbial Pathogenesis and Immunology, Texas A&M University Health Science Center, 3107 Medical Research & Education Building, 8447 State Highway 47, Bryan, TX 77807, USA

**Keywords:** autoantibody, myelin oligodendrocyte glycoprotein, autoimmune disease, demyelinating disease, Fc fusion proteins, antibody engineering, therapy

## Abstract

Current treatments for antibody-mediated autoimmunity are associated with lack of specificity, leading to immunosuppressive effects. To overcome this limitation, we have developed a class of antibody-based therapeutics for the treatment of autoimmunity involving antibodies that recognize the autoantigen, myelin oligodendrocyte glycoprotein (MOG). These agents (“Seldegs,” for selective degradation) selectively eliminate antigen (MOG)-specific antibodies without affecting the levels of antibodies of other specificities. Seldeg treatment of mice during antibody-mediated exacerbation of experimental autoimmune encephalomyelitis by patient-derived MOG-specific antibodies results in disease amelioration. Consistent with their therapeutic effects, Seldegs deliver their targeted antibodies to Kupffer and liver sinusoidal endothelial cells that are known to have tolerogenic effects. Our results show that Seldegs can ameliorate disease mediated by MOG-specific antibodies and indicate that this approach also has the potential to treat other autoimmune diseases where the specific clearance of antibodies is required.

## Graphical Abstract

**Figure:**
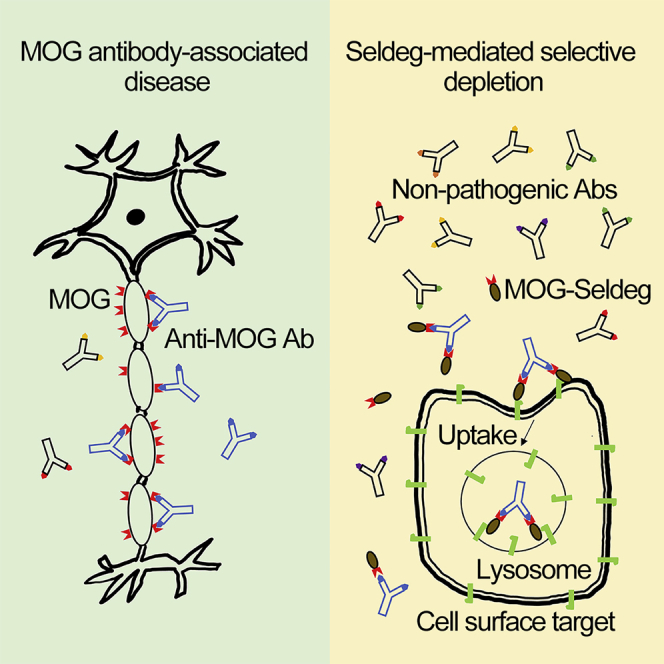

## Teaser

In this study, Sun and colleagues describe a strategy to selectively remove MOG-specific antibodies that cause autoimmunity, without affecting the levels of protective antibodies of other specificities. This approach has broad potential applications for the treatment of autoimmune diseases where the specific clearance of antibodies is desirable.

## Introduction

Although antibodies play a pivotal role in defense against pathogens, self-reactive antibodies contribute to the pathogenesis of autoimmunity in a plethora of diseases that include demyelinating and other neurological disorders.^1^, ^2^, ^3^ The binding of autoreactive antibodies to their target antigen can not only inhibit or activate cellular signaling pathways but can also lead to tissue damage through effector functions involving FcγRs and complement combined with antigen presentation and T cell activation.^3^ Despite the widespread occurrence of autoimmunity involving self-reactive antibodies, current treatments include the use of corticosteroids, the B cell depleting antibody rituximab and intravenous gammaglobulin (IVIg) that are not specific and can result in severe side effects.^4^, ^5^, ^6^ Plasma exchange represents an alternative treatment option, but this procedure is invasive and can also be associated with potentially fatal adverse events.^7^ There is therefore a pressing need for the development of targeted therapies that are more selective and do not have broad immunosuppressive effects. In the current study we describe the development of a therapeutic approach that selectively targets antibodies specific for myelin oligodendrocyte glycoprotein (MOG) without affecting the levels of (protective) antibodies of other specificities. This autoantigen is exposed on the outer myelin sheath^8^ and represents a target for autoantibodies in demyelinating disease.^2^^,^^9^, ^10^, ^11^, ^12^

Extensive analyses have been carried out in animal models to investigate the contribution of MOG-specific antibodies to the pathogenesis of disease.^13^, ^14^, ^15^, ^16^, ^17^, ^18^ Studies in rodent models have demonstrated that this protein is a target of autoreactive B and T cell responses that result in experimental autoimmune encephalomyelitis (EAE) and neuromyelitis optica.^13^^,^^14^^,^^18^^,^^19^ In addition, the implementation of assays for the detection of MOG-specific antibodies that recognize MOG in its native conformation^2^^,^^9^^,^^19^^,^^20^ has indicated the involvement of MOG-specific antibodies in the pathogenesis of multiple demyelinating diseases that include acute disseminated encephalomyelitis (ADEM), anti-aquaporin-4-antibody-seronegative neuromyelitis optica spectrum disorder (NMOSD), myelitis, optic neuritis, and brainstem encephalitis.^2^^,^^10^, ^11^, ^12^ MOG antibody-associated disease (MOGAD) affects both children and adults, can lead to severe neurological dysfunction, and frequently has a relapsing course.^10^^,^^12^^,^^21^ Further, reports that multiple sclerosis (MS) patient-derived antibodies specific for native MOG (human and rodent) can exacerbate EAE in rodents,^17^^,^^22^ combined with histopathological analyses demonstrating antibody and complement deposits in the CNS for over 50% of MS patients,^23^ indicate that such antibodies can also contribute to pathology in this potentially devastating disease. Collectively, the developments in characterizing MOG-specific responses have led to a paradigm shift in the understanding of how antibody responses are key players in demyelinating disease. Importantly, in addition to direct effects of MOG-specific antibodies in damaging the myelin sheath, such antibodies can enhance antigen presentation by central nervous system (CNS)-resident or peripheral antigen-presenting cells to autoreactive T cells.^24^^,^^25^

In the current study, we have investigated the therapeutic efficacy of engineered MOG-Fc fusion proteins in a mouse model of MS. These fusion proteins, named Seldegs (for selective degradation), are designed to rapidly and selectively deplete antigen (MOG)-specific antibodies by binding to both cell surface molecules (targeting component; to enable cellular uptake) and antigen-specific antibodies (antigen component; to capture specific antibodies). To explore the effects of targeting different cell surface molecules using Seldegs, we have generated an Fc fusion in which the targeting component binds to exposed phosphatidylserine (PS) on cells and compared this with a Seldeg that binds to the internalizing receptor, FcRn.^26^ The mouse model that we have used involves the transfer of MOG-specific antibodies derived from the serum of MS patients into mice following induction of mild T cell-mediated EAE, leading to antibody-mediated exacerbation of disease.^17^ We demonstrate that both Seldeg formats are effective in ameliorating antibody-mediated disease in this model. Further, by contrast with strategies such as the use of FcRn inhibitors that lower the concentrations of IgGs of all specificities,^27^, ^28^, ^29^, ^30^ Seldegs do not affect the levels of antibodies that are not specific for MOG. Our analyses provide support for the use of Seldegs, that avoid the generally immunosuppressive effects of current treatments, to treat demyelinating diseases involving MOG recognition. More generally, these engineered Fc fusions could have applications in clinical situations where the targeted depletion of antigen-specific antibodies is required.

## Results

### Generation of a PS-Targeting Seldeg

**Figure 1:**
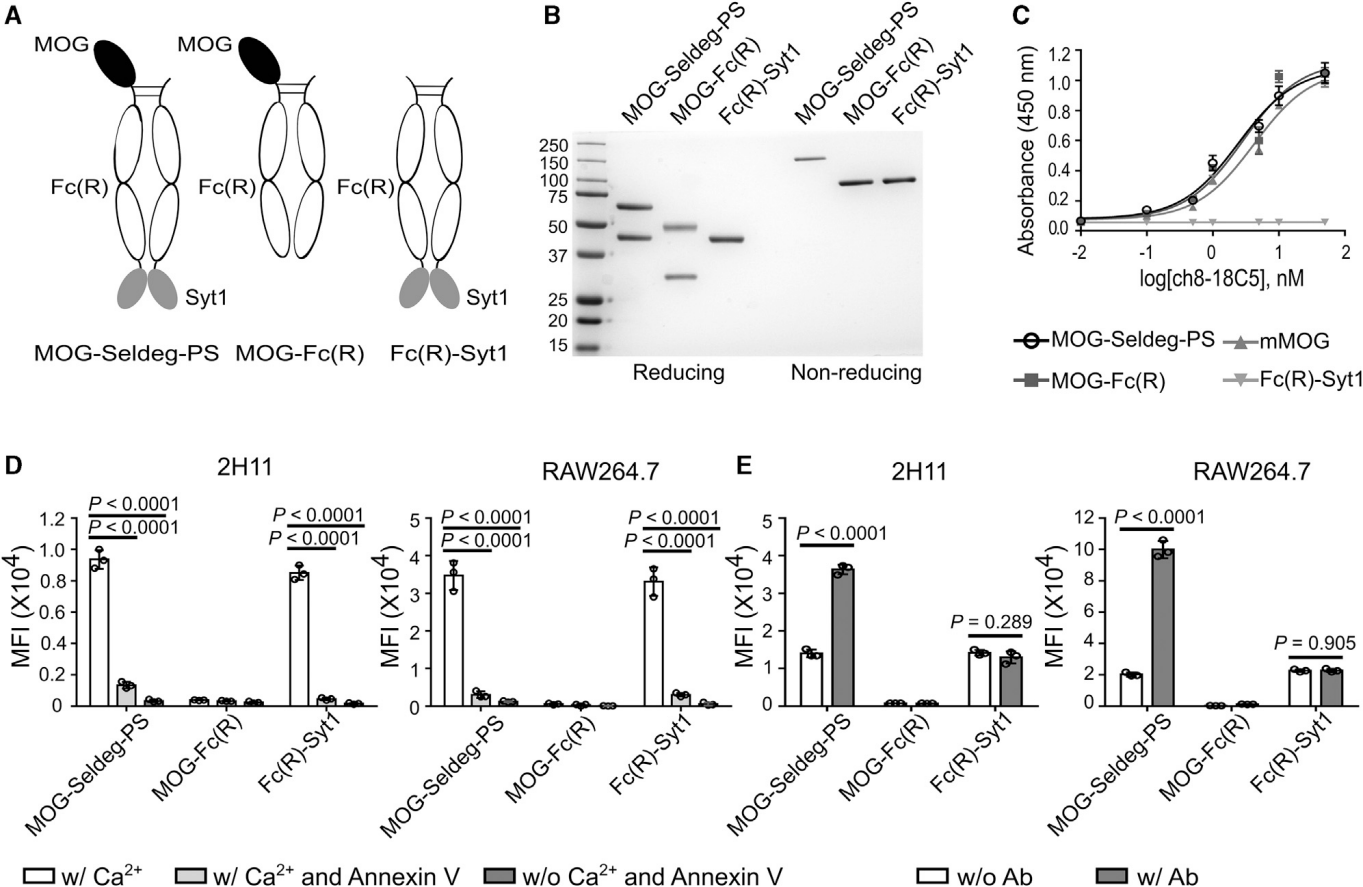
Generation and Characterization of MOG-Seldeg-PS and Control Fc Fusions (A) Schematic representation of MOG-Seldeg-PS, MOG-Fc(R), and Fc(R)-Syt1. (B) SDS-PAGE analysis of MOG-Seldeg-PS, MOG-Fc(R), and Fc(R)-Syt1. Proteins were analyzed under reducing and non-reducing conditions. Size standards are shown in kDa on the left margin. (C) ELISA of binding of MOG-specific antibody, ch8-18C5 (a chimeric human IgG1/mouse antibody) to the Fc fusions, and mouse MOG. Bound ch8-18C5 was detected using HRP-conjugated antibody specific for human Fab. Averages of triplicate samples are shown and error bars represent SD. (D) Flow cytometry analyses of binding of Fc fusions to PS-positive 2H11 and RAW264.7 cells. Cells were pre-treated with or without 50 μg/mL Annexin V (to inhibit PS binding) or 2.5 mM calcium chloride prior to incubation with 10 nM Fc fusions on ice. Bound Fc fusions were detected using Alexa 647-labeled antibody specific for human IgG. Data shown are averages of mean fluorescence intensity (MFI) values for triplicate samples. Error bars indicate SD. Statistically significant differences were analyzed using one-way ANOVA with Tukey’s multiple comparison test. (E) Flow cytometry analyses of binding of Fc fusions to PS-positive 2H11 and RAW264.7 cells in the presence or absence of ch8-18C5 (“Ab”). 100 nM Alexa 647-labeled Fc fusions were pre-mixed with 25 nM ch8-18C5 before incubating with cells on ice. Averages of MFI values for triplicate samples are shown. Error bars indicate SD. Statistically significant differences were analyzed using unpaired Student’s t test (two-tailed). Data shown are representative of at least two independent experiments.

For comparison with a Seldeg that targets FcRn,^26^ we have generated a Seldeg format that binds to exposed PS on the surface of cells by fusing the extracellular domain of MOG to the N terminus of human IgG1-derived Fc, with the C2A domain of synaptotagmin 1 (Syt1) linked to the C terminus of the Fc. Binding to FcγRs was ablated by inserting G236R/L428R (“R”) mutations.^31^ The Fc fusion (MOG-Seldeg-PS) includes the use of knobs-into-holes^32^^,^^33^ and electrostatic steering mutations^34^ in the CH3 domains of Fc to enable monomeric display of MOG (Figure 1A). Analogous constructs encoding MOG-Fc(R) or Fc(R)-Syt1, that lack Syt1- or MOG-encoding genes, respectively, were produced as controls (Figure 1A; note that Fc(R)-Syt1 is homodimeric and consequently lacks the knobs-into-holes and electrostatic steering mutations). Following purification, SDS–PAGE and size exclusion analyses demonstrate that the Fc fusions can be purified without aggregation (Figures 1B and S1), and are stable following storage at 4°C (30 days) or 37°C (5 days) in PBS or human serum (Figure S2).

Analyses using enzyme-linked immunosorbent assays (ELISAs) demonstrated that MOG-Seldeg-PS binds specifically to the MOG-specific antibody ch8-18C5 (chimeric 8-18C5 containing mouse variable domains derived from the 8-18C5 hybridoma^35^ fused to human IgG1/κ constant regions; Figure 1C) and to cells that expose PS on their surface (Figures 1D and S3). The mouse tumor endothelial cell line, 2H11, and mouse macrophage cell line, RAW264.7, that both have cell surface exposed PS,^36^^,^^37^ were used for these analyses. The Fc-Syt1 fusions (MOG-Seldeg-PS and Fc(R)-Syt1) bound to these cells, and these interactions could be blocked by preincubation with the PS-binding protein, Annexin V (Figure 1D). Consistent with previous reports,^36^ the binding of the Syt1-containing fusions was also Ca^2+^-dependent (Figure 1D). Furthermore, the surface binding of MOG-Seldeg-PS to both 2H11 and RAW264.7 cells increased in the presence of ch8-18C5 (Figures 1E and S3), most likely due to the ability of the MOG-specific antibody to dimerize MOG-Seldeg-PS and increase its binding avidity. Collectively, the data show that MOG-Seldeg-PS binds specifically to PS on the cell surface.

### MOG-Seldeg-PS Internalizes MOG-Specific Antibodies

**Figure 2:**
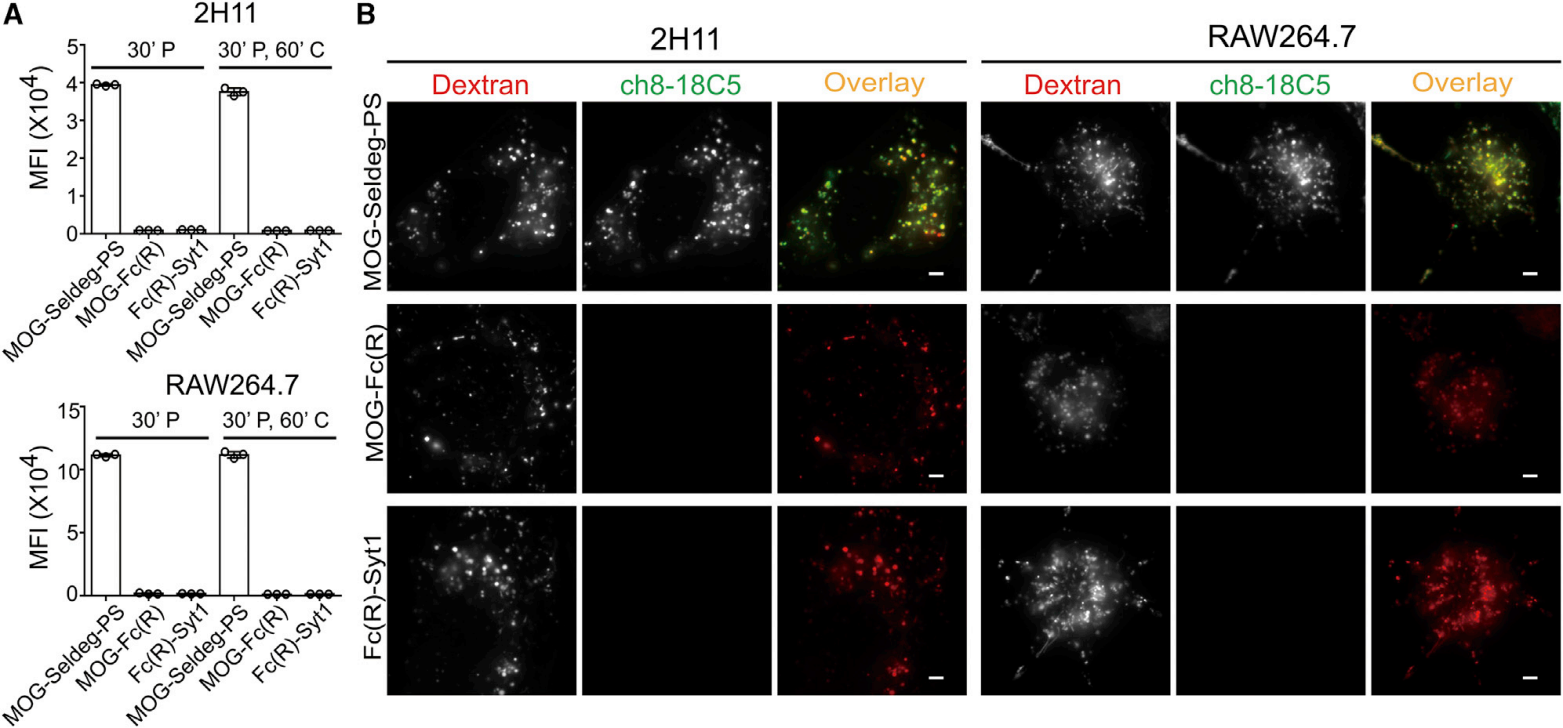
MOG-Seldeg-PS Increases the Accumulation of MOG-Specific Antibody in PS-Positive Cells (A) 2H11 and RAW264.7 cells were pulsed with 100 nM Alexa 647-labeled ch8-18C5 mixed with 400 nM MOG-Seldeg-PS, MOG-Fc(R), or Fc(R)-Syt1 for 30 min and chased for 0 min (30’ P) or 60 min (30’ P, 60’ C) at 37°C. Averages of MFI values for Alexa 647-labeled ch8-18C5 for triplicate samples are shown. Error bars indicate SD. (B) 2H11 and RAW264.7 cells were pre-pulsed with 100 μg/mL Alexa 555-labeled dextran for 3 h, washed, and chased for 3 h. Cells were then pulsed with 100 nM Alexa 647-labeled ch8-18C5 in complex with 400 nM of MOG-Seldeg-PS, MOG-Fc(R), or Fc(R)-Syt1 for 3 h followed by washing and a 3 h chase period. For the overlay images, Alexa 555 and Alexa 647 are pseudocolored red and green, respectively. Scale bars, 3 μm. Images shown are representative cells (n ≥ 34). Data shown are representative of at least two independent experiments.

To investigate the cellular behavior of the target antibody, ch8-18C5, in the presence of MOG-Seldeg-PS, we assessed the internalization and accumulation of ch8-18C5 in 2H11 and RAW264.7 cell lines using flow cytometry and fluorescence microscopy (Figures 2A and 2B). Incubation of cells with ch8-18C5 premixed with MOG-Seldeg-PS, followed by washes with calcium-free buffer (to remove surface bound Seldeg) resulted in accumulation of high levels of ch8-18C5 within both cell types (Figures 2A and S3). The majority of the internalized antibody (>95%) was retained within the cells during a 60-min chase period (Figure 2A). By contrast, ch8-18C5 accumulation within cells was at close to background fluorescence levels in the presence of the control Fc fusions, MOG-Fc(R), and Fc(R)-Syt1 (Figure 2A). Fluorescence microscopy was also used to analyze the intracellular fate of the targeted antibody, ch8-18C5. Cells were pulse-chased with fluorescently labeled dextran to label lysosomes, and following 3 h incubation with MOG-Seldeg-PS:ch8-18C5 complexes followed by a 3 h chase period, fluorescently labeled ch8-18C5 was present in the lysosomes (Figure 2B). Importantly, consistent with the flow cytometry data, ch8-18C5 was at undetectable levels in lysosomes following incubation of this antibody with cells in the presence of MOG-Fc(R) or Fc(R)-Syt1 (Figure 2B). These analyses demonstrate that MOG-Seldeg-PS captures and internalizes ch8-18C5 into the endolysosomal pathway within cells.

### MOG-Seldeg-PS Clears MOG-Specific Antibody *In Vivo*

**Figure 3:**
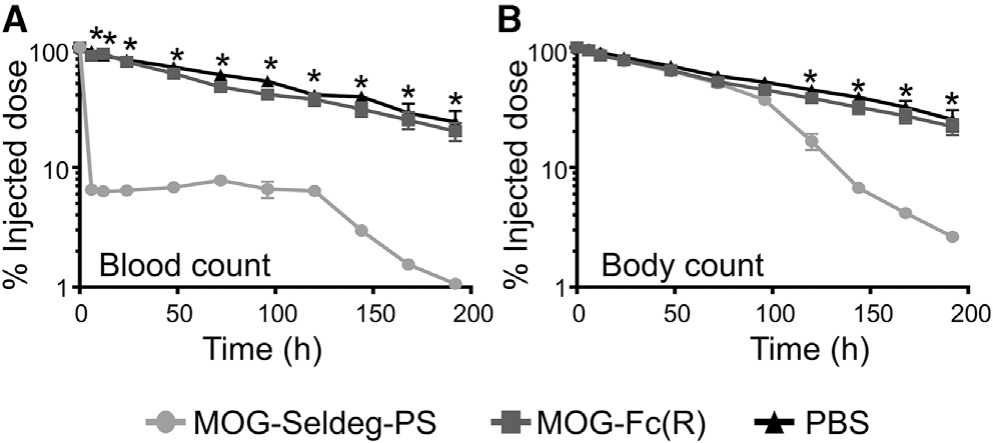
The MOG-Specific Antibody, ch8-18C5, Is Rapidly Cleared by MOG-Seldeg-PS in Mice Transgenically Expressing huFcγRs Mice were intravenously injected with radiolabeled (125-I) ch8-18C5 (15 μg) and 24 h later (0 time point on plots) injected with a 4-fold molar excess of MOG-Fc(R) (31 μg), MOG-Seldeg-PS (40 μg), or, as control, PBS (n = 5 mice/group). (A and B) Radioactivity levels in blood (A) or whole body (B) were determined at the indicated times. The radioactivity levels obtained immediately before MOG-Seldeg-PS or control delivery were taken as 100%. Error bars indicate SEM and statistically significant differences are indicated for MOG-Fc(R) versus MOG-Seldeg-PS by ∗ (p < 0.0001, two-way analysis of variance with Tukey’s multiple comparisons). Data shown are representative of at least two independent experiments.

We next assessed the ability of MOG-Seldeg-PS to increase the clearance rate of the MOG-specific antibody, ch8-18C5, in mice that transgenically express human FcγRs (huFcγR mice^38^). These mice were used since the Fc fusions and ch8-18C5 comprise human IgG1-derived Fc regions. Mice were injected with ^125^I-labeled, MOG-specific antibody ch8-18C5, and 24 h later, MOG-Seldeg-PS or control protein (MOG-Fc(R)) was injected at a 4-fold molar excess over the target antibody. The delivery of MOG-Seldeg-PS resulted in a rapid decrease in ch8-18C5 levels in the blood compared with that observed in control (MOG-Fc(R) and PBS) groups (Figure 3). Increased clearance of ch8-18C5 was also observed in the presence of MOG-Seldeg-PS relative to controls at the whole-body level, except that the start of the clearance was delayed (Figure 3). Serum levels of ch8-18C5 were decreased to ∼6%–7% injected dose within 6 h of Seldeg delivery. Importantly, the total IgG levels in serum of mice prior to and following treatment with MOG-Seldeg-PS were not significantly different (Figure S4), indicating the selectivity of MOG-Seldeg-PS mediated clearance.

**Figure 4:**
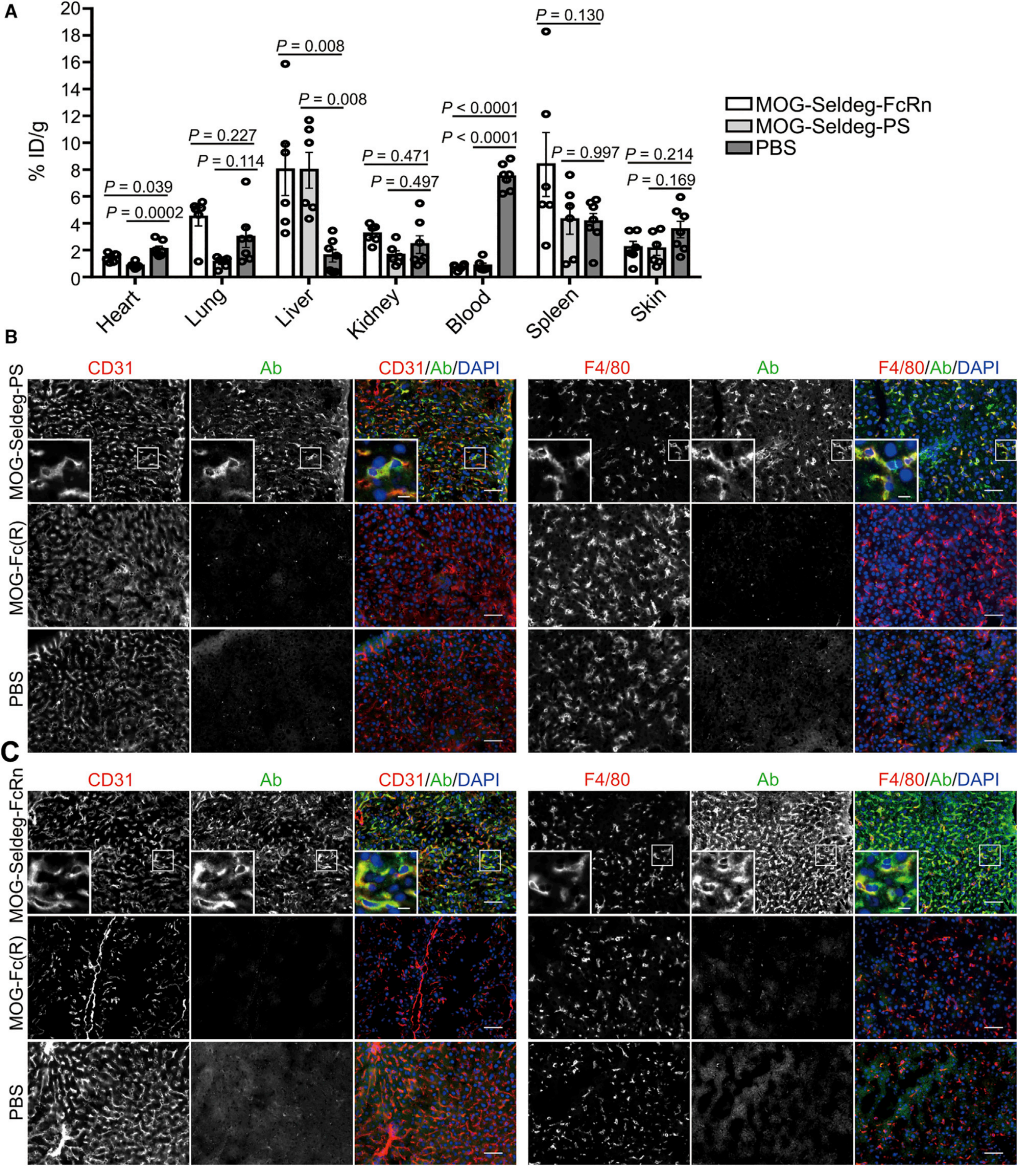
MOG-Specific Antibody Shows Increased Levels in Liver Cells following Seldeg Treatment of Mice (A–C) Transgenic mice expressing huFcγRs were i.v. injected with radiolabeled (125-I) or unlabeled ch8-18C5 (15 μg) in (A) or (B) and (C), respectively. 24 h following ch8-18C5 delivery, mice were i.v. injected with MOG-Seldeg-FcRn (31 μg), MOG-Seldeg-PS (40 μg), or as controls, MOG-Fc(R) (31 μg) or PBS. Blood and various organs were harvested 6 h following Seldeg or control delivery (equivalent to 30 h after injection of ch8-18C5). (A) Biodistribution of 125-I labeled ch8-18C5 in mice (n = 6 mice/group, from two duplicate experiments with 3 mice/group; percentage injected dose per gram [% ID/g]). Error bars indicate SEM and statistically significant differences are indicated for each Seldeg versus PBS by one-way ANOVA with Tukey’s multiple comparison test. (B and C) Colocalization of ch8-18C5 (pseudocolored green in overlays) with CD31^+^ endothelial cells or F4/80^+^ macrophages (pseudocolored red in overlays) in liver following treatment of mice with MOG-Seldeg-PS (B) or MOG-Seldeg-FcRn (C). DAPI is pseudocolored blue in the overlays. Representative cells (boxed) are cropped and expanded in the panels on the left-hand side. Scale bars, 50 μm, and for expanded images, scale bars, 10 μm. Data shown is derived from immunohistochemical analyses of one mouse per group, with 12–59 images acquired per tissue. Data shown are representative of two independent IHC experiments.

We next analyzed the biodistribution of the MOG-specific antibody ch8-18C5 following the delivery of MOG-Seldeg-PS. As a comparator, we also used a Seldeg (MOG-Seldeg-FcRn) that comprises MOG linked to a heterodimeric, mutated human IgG1-derived Fc fragment.^26^ The mutated Fc is engineered to bind to FcRn with increased affinity in the pH range 6–7.4, conferring an ability of this Seldeg to internalize and target MOG-specific antibodies to lysosomes in cells and induce the rapid clearance of such antibodies in mice.^26^ Mice were injected with ^125^I-labeled ch8-18C5, followed by MOG-Seldeg-PS, MOG-Seldeg-FcRn, or, as controls, MOG-Fc(R) or PBS. Following 6 h of treatment, organs were harvested and radioactivity levels determined. By comparison with mice in control vehicle (PBS)-treated groups, the level of radiolabel was 3- to 4-fold higher in the liver for mice treated with the Seldegs, indicating that the liver is a major site of Seldeg-mediated delivery of ch8-18C5 (Figure 4A). The behavior of ch8-18C5 in biodistribution analyses was similar in the presence of vehicle control (PBS) or control Fc fusion, MOG-Fc(R) (Figure S5). Consistent with the clearance studies (Figure 3),^26^ the level of radiolabel in blood was substantially lower in mice treated with Seldegs relative to controls at 6 h post-treatment.

The observation of efficient Seldeg-mediated delivery of MOG-specific antibody, ch8-18C5, to the liver in biodistribution analyses was followed by immunohistochemistry (IHC) to investigate the distribution and localization of the targeted antibody at the cellular level in this organ (Figures 4B and 4C). Mice were treated with unlabeled ch8-18C5 followed by a 4-fold molar excess of Seldeg, or as controls, MOG-Fc(R) or PBS. Following 6 h treatment, livers were harvested and IHC carried out, using antibodies specific for F4/80 and CD31 to detect Kupffer cells and liver sinusoidal endothelial cells (LSECs), respectively. To distinguish ch8-18C5 from Fc fusions or endogenous mouse IgG, we used a fluorescently labeled human Fab-specific antibody to detect ch8-18C5 antibody. These studies revealed that in the presence of either Seldeg, ch8-18C5 was associated with both LSECs and Kupffer cells (Figures 4B and 4C). Consistent with the biodistribution analyses, ch8-18C5 levels were substantially lower in the liver following treatment with MOG-Fc(R) or PBS (Figures 4B and 4C).

### Seldeg Treatment Ameliorates Antibody-Mediated EAE

**Figure 5:**
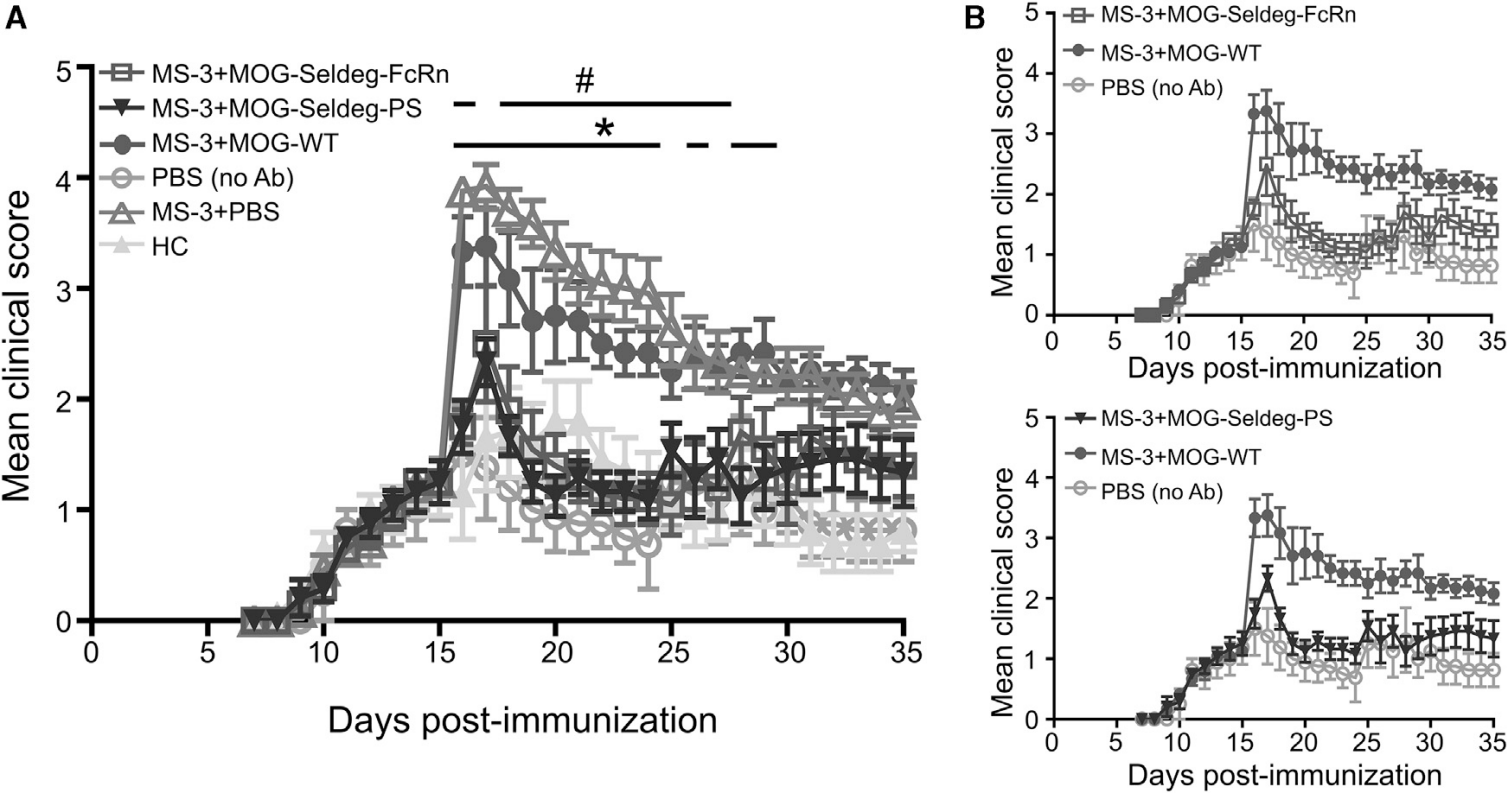
Seldeg Treatment Ameliorates Disease in Mice with EAE huFcγR mice (n = 4–6 mice/group) were immunized with hMOG35-55, and 15 days following immunization, mice were injected i.v. with 250 μg MS patient-derived IgG (MS-3), 250 μg healthy control (HC)-derived IgG, or with PBS (vehicle; “no Ab”). 2 h later, mice were i.v. injected with 50 μg MOG-Seldeg-PS, MOG-Seldeg-FcRn, MOG-WT^26^ (comprising a heterodimeric MOG-Fc fusion with knobs-into-holes mutations and G236R/L328R mutations), or PBS (vehicle). Mice were scored daily for disease activity. (A) Mean disease scores for each treatment group. (B) To facilitate visualization of disease course, selected datasets derived from (A) showing treatment with MOG-Seldeg-PS or MOG-Seldeg-FcRn versus controls are shown. Error bars indicate SEM and statistically significant differences between the groups are indicated by # and ∗ (p < 0.05; two-way ANOVA and Tukey’s multiple comparison test). #p values for MOG-Seldeg-FcRn versus MOG-WT, with corresponding day in parentheses: p = 0.0001 (16), 0.0088 (18), 0.0113 (19), 0.0015 (20), 0.0008 (21), 0.0015 (22), 0.0022 (23), 0.0022 (24), 0.0075 (25), 0.0244 (26), 0.021 (27); ∗p values for MOG-Seldeg-PS versus MOG-WT, with corresponding day in parentheses: p = 0.0001 (16), 0.0209 (17), 0.0003 (18), 0.0002 (19), 0.0001 (20), 0.0003 (21), 0.0009 (22), 0.0023 (23), 0.0009 (24), 0.0139 (26), 0.0014 (28), 0.0091 (29). Data are representative of two independent experiments.

An antibody-mediated EAE disease model that we have described previously^17^ was used to investigate whether Seldeg treatment has therapeutic effects. This EAE model involves the transfer of MOG-specific antibodies isolated from sera of MS patients into huFcγR mice^38^ that have been immunized with the weakly encephalitogenic human MOG peptide (residues 35–55; hMOG35-55).^14^^,^^17^ These patient-derived antibodies have been shown in our earlier studies to cross-react with mouse MOG, and hMOG35-55 peptide immunization results in mild disease that is exacerbated by antibody transfer.^17^ On day 15 following peptide immunization, when the mean clinical score in each group had reached ∼1–2, mice were treated with purified IgG derived from an MS patient (MS-3) or healthy control (HC) followed by the delivery of Seldegs or controls (MOG-WT^26^ or PBS vehicle; Figure 5). Delivery of either MOG-Seldeg-PS or MOG-Seldeg-FcRn following antibody transfer resulted in substantial reductions of the antibody-mediated exacerbation of disease that persisted until the experimental endpoint (20 days post-treatment) compared with disease activity observed for mice in control groups (treated with MOG-WT or PBS vehicle; Figure 5). Importantly, the disease activity in the Seldeg treatment groups was not significantly different to that in groups of mice that had been injected with IgG from a HC or PBS instead of patient-derived IgG. In addition, the therapeutic effect was observed within 1 day of treatment and there was no significant difference in mean disease scores between groups treated with FcRn- or PS-targeting Seldegs.

## Discussion

MOG-specific antibodies play a central role in the pathogenesis of MOGAD that includes ADEM, anti-aquaporin-4-antibody-seronegative NMOSD, myelitis, and optic neuritis.^2^^,^^10^, ^11^, ^12^ These demyelinating diseases frequently follow a relapsing course, resulting in debilitating effects that can lead to death.^10^^,^^12^^,^^21^ Current therapies for MOGAD and other antibody-mediated autoimmune diseases involve agents with broad immunosuppressive activities such as B cell depletion, corticosteroids, or IVIg.^2^^,^^10^^,^^39^^,^^40^ These treatments can be associated with serious side effects including increased risk of infection.^5^^,^^6^ In addition, the use of plasma exchange as an alternative treatment is highly invasive and can result in life-threatening consequences such as thrombosis.^7^ These non-specific therapies do not specifically target autoreactive antibodies that are the underlying cause of disease. The development of a non-invasive approach to specifically remove these antibodies, while leaving the remaining immune repertoire intact, therefore represents an attractive possibility that would overcome the disadvantages of available treatments.

To address the limitations of broadly immunosuppressive treatments, we have generated engineered Fc fusions (Seldegs) that selectively and rapidly deplete autoreactive antibodies without affecting antibodies of other antigen specificities. Seldegs comprise a targeting, engineered Fc component that binds to cell surface receptors or molecules and an antigen component that captures specific antibodies. We have shown that two different formats of Seldegs are effective in the selective depletion of antibodies that recognize MOG, while not affecting total mouse IgG levels. The Seldegs also ameliorate disease exacerbation mediated by human MS patient-derived, MOG-specific antibodies in a mouse model of MS. Importantly, the therapeutic effects persist for up until 20 days post-treatment, and the disease activity in treated mice is analogous to that in mice in control groups that were not subjected to disease exacerbation.

Fc fusions comprising Fc fragments linked to different proteins are widely used as therapeutics in the clinic and have favorable safety profiles.^41^ Further, even in the presence of (polyclonal) autoantibodies that recognize the protein that is linked to the Fc, such fusion proteins have tolerizing effects through mechanisms that relate to, for example, the pharmacokinetic behavior of the Fc fusion and/or the presence of tolerogenic epitopes (Tregitopes).^42^^,^^43^ Internalization of “free” Seldegs without bound antigen-specific antibody through FcRn- or PS-mediated interactions could occur when these agents are delivered in excess over their target antibodies or fail to capture such antibodies. Based on our earlier analyses of the subcellular trafficking behavior of Fc-Syt1 fusions or Fc fragments with increased FcRn binding,^29^^,^^36^^,^^44^ in combination with the tolerizing effects of Fc fusions and, for MOG-Seldeg-PS, PS-mediated internalization,^42^^,^^43^^,^^45^ we expect that this will not lead to adverse consequences. The antigen component of the MOG-specific Seldegs is relatively small, comprising the single Ig-like, extracellular domain of MOG of 117 amino acids. MOG-specific antibodies have been shown by us and others to compete with the monoclonal anti-MOG antibody, 8-18C5, for binding in the majority of patients, indicating that they are directed toward a specific region of MOG that, based on the binding specificity of 8-18C5, encompasses or is proximal to the FG-loop.^17^^,^^46^ The construction of Seldegs with one antigen molecule per Fc (hetero)dimer is therefore expected to result in complexes with stoichiometries of one MOG-specific antibody per one or two Seldeg molecules, rather than highly multimeric immune complexes that may result in inflammation. Importantly, we demonstrate that Seldegs efficiently target MOG-specific antibodies to LSECs and Kupffer cells, which have tolerizing properties through pathways involving tolerogenic priming or induction of regulatory T cells.^47^^,^^48^ The amelioration of MOG-specific antibody-mediated exacerbation of EAE by Seldegs is consistent with this targeting behavior and observations of others using Fc fusions.^42^^,^^43^

Two Seldegs that target different surface molecules (FcRn and PS) have been used in the current study. Delivery of either of these Seldegs results in similar elimination behavior of the targeted antibody from the circulation, with the majority (around 80%–95%) being cleared within several hours of delivery (this study and our earlier analyses^26^). This fast clearance is expected to confer significant advantages, particularly for autoimmune diseases where onset can occur rapidly and even be life-threatening. For the FcRn-targeting Seldeg, the rapid internalization of FcRn, combined with our earlier studies, indicates that the predominant clearance pathway is through internalization into the endolysosomal pathway in FcRn-expressing cells.^26^ Our recent analyses have indicated that FcRn is very active in highly pinocytic cells such as macrophages/Kupffer cells.^49^ This activity, combined with the reduced blood flow rate through the sinusoids, results in high exposure of circulating molecules to both Kupffer cells and LSECs,^50^ and is consistent with the efficient delivery of MOG-specific antibodies to these cells by the FcRn-targeting Seldeg. By contrast with the FcRn-targeting Seldeg, the *in vivo* clearance pathways for the PS-targeting Seldeg appear to be more complex, with rapid clearance of iodinated MOG-specific antibody from the circulation but substantially slower whole-body clearance. This suggests that in addition to delivery of Seldeg:antibody complex to lysosomes following internalization into the endolysosomal pathway, the complexes bind to a subset of PS-positive cells, which are characterized by slow PS internalization and/or lysosomal delivery. Further, although PS-positive cells are typically engulfed by phagocytic cells such as macrophages and Kupffer cells via non-inflammatory processes,^45^^,^^50^ which could result in Seldeg-mediated clearance via an indirect pathway, PS exposure has been reported to be necessary but not sufficient for this process.^51^ Consequently, it is conceivable that relatively long-lived, PS-exposing cell types confer slow whole-body clearance by acting as carriers of PS-targeting Seldegs *in vivo*. Nevertheless, although there are differences in the dynamic behavior of targeted antibody in the presence of FcRn- and PS-targeting Seldegs, both formats rapidly remove circulating levels of pathogenic, MOG-specific antibodies, and ameliorate disease.

In earlier analyses using a similar model of antibody-mediated exacerbation of EAE to that used in the current study, we demonstrated that passively transferred MOG-specific antibody (8-18C5) can be detected in brain sections of mice within 2 h of transfer.^52^ Consequently, the ability of Seldegs to ameliorate disease when they are delivered 2 h post-antibody transfer indicates that these agents can deplete targeted antibodies in both the CNS and periphery. Related to this, recent analyses in humans have shown that by analogy with the dominance of extrathecal production of aquaporin-4-specific antibodies in NMOSD, intrathecal synthesis of IgG is infrequent in MOGAD.^53^^,^^54^ This suggests that the peripheral depletion of MOG-specific antibodies in MOGAD will be an important contributor to the potential therapeutic effects of Seldegs.

In summary, we have shown that Seldegs are effective in treating MOG-specific antibody-mediated exacerbation in murine EAE through their ability to efficiently and specifically clear antigen-specific antibodies. These studies indicate that the depletion of MOG-specific antibodies with Seldegs could provide a targeted therapeutic approach for potentially devastating diseases such as ADEM and NMOSD. Finally, the Seldeg approach has the potential to have broad applications as a highly specific treatment for other antibody-mediated autoimmune diseases.

## Materials and Methods

### Cell Lines and Culture Conditions

The mouse tumor-derived endothelial cell line, 2H11 (ATCC, CRL-2163, Manassas, VA, USA), and the mouse macrophage cell line, RAW264.7 (ATCC, TIB-71), were cultured in Dulbecco’s modified Eagle’s medium (DMEM) supplemented with 10% fetal bovine serum (FBS). Cells were cultured at 37°C with 5% CO_2_. Expi293F cells (Thermo Fisher Scientific, Waltham, MA, USA) were cultured in Expi293 expression medium (Thermo Fisher Scientific) at 37°C with 8% CO_2_/80% humidity with shaking at 120 rpm. Cell lines were tested at monthly intervals for mycoplasma contamination and were authenticated annually at the University of Arizona Genetics Core through DNA fingerprint analysis.

### Human Serum Samples

200–300 μL serum from an MS patient (labeled MS-3) or from a HC were diluted with phosphate buffered saline and IgG was purified using protein G-Sepharose (GE Healthcare, Chicago, IL, USA). Serum samples were collected from MS patients and HCs under a UT Southwestern IRB approved biorepository (STU022011-211) and de-identified before being provided to the laboratory.

### Generation of Expression Constructs

The expression vector pcDNA3.4-TOPO (Invitrogen, Carlsbad, CA, USA) was used for expression of all constructs in this study. In addition, for all Fc fusion constructs, the cysteine (C220) in the IgG1 hinge region that bridges with cysteine in the light chain constant region was mutated to serine. The generation of the expression construct for MOG-Seldeg-FcRn has been described previously.^26^ For MOG-Seldeg-PS, two constructs comprising human synaptotagmin I (Syt1) C2A domain (residues 141-266) linked to the C terminus of the human IgG1 Fc fragment^36^ with insertion of knobs-into-holes mutations KiH1 (S364H/F405A) or KiH2 (Y349T/T394F)^32^^,^^33^ were generated. The gene encoding the Fc-Syt1 (KiH2) fusion was then spliced through the hinge region to a gene encoding the extracellular domain of mouse MOG (codons 1–117) via a Gly_4_Ser linker using splicing by overlap extension^55^ to generate MOG-Fc-Syt1 (KiH2). Mutations to ablate binding to FcγRs (G236R/L328R^31^) were inserted into Fc-Syt1 (KiH1) and MOG-Fc-Syt1 (KiH2), and these constructs were subsequently modified by insertion of electrostatic steering mutations E357K/D399K and K392D/K409D,^34^ respectively. The resulting MOG-Fc-Syt1 and Fc-Syt1 fusions were co-expressed to generate MOG-Seldeg-PS.

For the control heterodimeric protein, MOG-Fc(R), for MOG-Seldeg-PS, constructs for the expression of a heterodimeric MOG-Fc fusion (MOG-WT,^26^ MOG fused to human IgG1 Fc with KiH2 mutations, human IgG1 Fc with KiH1 mutations, and G236R/L328R [“R”] mutations in both constructs) with insertion of electrostatic steering mutations (E357K/D399K for Fc, and K392D/K409D for MOG-Fc) were generated. These two constructs were used in co-transfections to express MOG-Fc(R). MOG-WT^26^ was also used as a control protein in disease experiments using MOG-Seldeg-PS and the previously described FcRn-targeting Seldeg, MOG-Seldeg-FcRn.^26^ A construct encoding a homodimeric protein, Fc(R)-Syt1, comprising human synaptotagmin I (Syt1) C2A domain (residues 141–266) linked to the C terminus of the human IgG1 Fc fragment with G236R/L328R (“R”) mutations^31^ in the Fc fragment, was generated for use as an additional control.

The plasmid constructs to express the heavy and light chain genes of a chimeric MOG-specific antibody 8-18C5^35^ (ch8-18C5) fused to the human IgG1-derived constant heavy (CH) and constant light (Cκ) chain domain genes, respectively, have been described previously.^17^ All constructs were generated using standard methods of molecular biology and designed oligonucleotides. Oligonucleotide and construct sequences are available upon request.

### Protein Expression and Purification

Recombinant mouse MOG (extracellular domain) was purified from the culture supernatant of baculovirally infected High -Five cells using Ni^2+^-NTA agarose (QIAGEN, Germantown, MD, USA) as described previously.^56^ All Fc fusion proteins and the ch8-18C5 antibody were expressed in Expi293F cells (Thermo Fisher Scientific) following transient transfection with the GIBCO Expi293 expression system kit (Thermo Fisher Scientific) and were purified from culture supernatants using protein G-Sepharose (GE Healthcare) or, for MOG-Seldeg-FcRn, protein A-Sepharose (Invitrogen). Recombinant proteins were eluted from the columns using 50 mM diethylamine/150 mM NaCl and immediately neutralized using 2 M Tris-HCl pH 7.0 followed by dialysis against phosphate-buffered saline (PBS). Recombinant Fc fusion proteins were further purified using a Hiload 16/600 Superdex 200 gel filtration column (GE Healthcare). Purified proteins were analyzed by SDS-PAGE and by size exclusion chromatography (SEC) using a Phenomenex Yarra 3 μm SEC-3000 column (Phenomenex, 00H-4513-K0, Torrance, CA, USA).

### Protein Labeling

Proteins were labeled with Alexa Fluor 647 NHS Ester (Thermo Fisher Scientific) following the manufacturer’s instructions. The ch8-18C5 antibody was fluorescently labeled with a dye to antibody molar ratio of 1.8. Fc fusion proteins were fluorescently labeled with dye to protein molar ratios of 0.9 (MOG-Seldeg-PS), 0.8 (MOG-Fc(R)) and 0.9 (Fc(R)-Syt1). Fluorophore-labeled proteins were analyzed using a Phenomenex Yarra 3 μm SEC-3000 column (Phenomenex, 00H-4513-K0).

Proteins were labeled with I-125 using Iodogen (Perkin Elmer, Covina, CA, USA or MP Biomedicals, Santa Ana, CA, USA) as described previously.^57^

### Flow Cytometry Analyses

For binding assays, 2H11 or RAW264.7 cells were harvested by trypsinization or pipetting, respectively, resuspended in flow cytometry buffer (10 mM HEPES, 140 mM NaCl, 1% bovine serum albumin [BSA], pH 7.4) with or without 1.5 mM CaCl_2_ and with or without 50 μg/mL Annexin V (BioLegend, San Diego, CA, USA) and incubated in flow cytometry tubes (∼0.5 × 10^6^ cells/tube) for 15 min at room temperature. Following 15 min incubation, 10 nM MOG-Seldeg-PS, MOG-Fc(R), or Fc(R)-Syt1 was added followed by incubation for 30 min on ice. Cells were washed twice with ice-cold flow cytometry buffer and incubated with goat anti-human IgG (H+L) antibody conjugated with Alexa Fluor 647 (Thermo Fisher Scientific) on ice for 30 min. Following washing with ice cold flow cytometry buffer, cells were analyzed using a BD Accuri C6 flow cytometer (BD Biosciences, Franklin Lakes, NJ, USA).

To quantitate the effect of the presence of ch8-18C5 antibody on the cell surface binding of MOG-Seldeg-PS, we harvested 2H11 or RAW264.7 cells as above and resuspended in binding buffer (10 mM HEPES, 140 mM NaCl, 1.5 mM CaCl_2_, 1% BSA, pH 7.4) in flow cytometry tubes. Cells were incubated with 100 nM Alexa Fluor 647 labeled MOG-Seldeg-PS, MOG-Fc(R), or Fc(R)-Syt1 in the presence or absence of 25 nM ch8-18C5 antibody for 30 min on ice. Cells were washed with ice cold binding buffer and analyzed using a BD Accuri C6 flow cytometer.

For recycling assays, 2H11 or RAW264.7 cells were plated in 24-well plates at a density of 50,000 cells per well. 24 h following plating, cells were pulsed with 100 nM Alexa Fluor 647-labeled ch8-18C5 antibody in complex with 400 nM MOG-Seldeg-PS, MOG-Fc(R) or Fc(R)-Syt1 for 30 min at 37°C. This pulse was followed by two washes with PBS at room temperature, followed by washing (0 min chase) or washing and chasing in medium for 60 min (60 min chase) at 37°C. At the end of the chase period, cells were washed with ice cold PBS and harvested by trypsinization (2H11) or pipetting (RAW264.7). Harvested cells were collected, washed with ice cold PBS, and analyzed using a BD Accuri C6 flow cytometer. All flow cytometry data were processed using FlowJo (FlowJo, Ashland, OR, USA).

### ELISAs

Purified mMOG, MOG-Seldeg-PS, MOG-Fc(R) or, as control, Fc(R)-Syt1 were coated on Polysorb microtiter plates (Nunc-Immuno, Thermo Fisher Scientific) at a concentration of 10 nM in PBS (pH 7.4) overnight at 4°C, washed with PBS and blocked with 2% milk powder/PBS followed by addition of serially diluted ch8-18C5 antibody at room temperature. Following incubation for 60 min, plate wells were washed four times with PBS containing 0.1% Tween, and bound antibodies were detected using goat anti-human IgG (Fab-specific) antibody conjugated with HRP (Sigma-Aldrich, St. Louis, MO, USA). 3,3′,5,5′-Tetramethylbenzidine (TMB) substrate (Acros Organics, Geel, Belgium) was added and the reaction stopped after 5 min. Absorbance was measured at 450 nm using a microplate reader (BioTek, Winooski, VT, USA).

### Analyses of Stability of MOG-Seldeg-PS

MOG-Seldeg-PS was incubated in PBS at 4°C for 30 days or 37°C for 5 days, followed by analyses using a Phenomenex Yarra 3 μm SEC-3000 column (Phenomenex, 00H-4513-K0). For serum stability assays, endogenous IgGs were depleted from human serum (Sigma-Aldrich) by passage through protein G-Sepharose (GE Healthcare). MOG-Seldeg-PS was incubated in serum at a concentration of 400 nM at 37°C for 3 or 5 days as described previously.^26^ Following incubation, proteins were immunoprecipitated using agarose beads coupled with goat anti-human Fc-specific antibody (Sigma-Aldrich). Bound proteins were detected by immunoblotting with HRP-conjugated goat anti-human IgG (H+L) antibody (Jackson ImmunoResearch, West Grove, PA, USA). The immunoreactive bands were detected using WesternSure substrate (Thermo Fisher Scientific), followed by scanning with a C-DiGit blot scanner (LI-COR, Lincoln, NE, USA).

### Fluorescence Microscopy Analyses

2H11 or RAW264.7 cells were plated in phenol red-free DMEM medium on micro-coverglasses (Electron Microscopy Sciences, Hatfield, PA, USA) and incubated overnight in a 37°C incubator with 5% CO_2_. To label lysosomes, we pre-pulsed cells with 100 μg/mL Alexa Fluor 555-labeled dextran (Thermo Fisher Scientific) for 3 h, followed by washing with PBS (room temperature) and a 3 h chase. Cells were then pulsed with 100 nM Alexa Fluor 647-labeled ch8-18C5 antibody in complex with 400 nM MOG-Seldeg-PS, MOG-Fc(R) or Fc(R)-Syt1 for 3 h, washed with PBS (room temperature) twice, and subsequently chased in medium for 3 h.

Cells were imaged with an Axio Observer Z1 inverted epifluorescent microscope (Zeiss, Oberkochen, Germany) equipped with a 63×, 1.4 NA Plan-Apochromat objective (Zeiss), and a Zeiss 1.6× internal optovar. Images were acquired with filter sets for Alexa Fluor 555 (Cy3-4040C-ZHE) and Alexa Fluor 647 (Cy5-4040C-ZHE) from Semrock using a Hamamatsu Orca ER CCD camera (Hamamatsu Photonics Systems, Model C4742-95-12ER, Hamamatsu-city, Japan). Acquired images were analyzed using in-house written software (MIATool) (www.wardoberlab.com/software/miatool).^58^ Acquired images for Alexa 647 were linearly adjusted with the same intensity adjustment settings.

### Mice

Mice that transgenically express human FcγRs^38^ were bred in a pathogen-free facility at Texas A&M University and experiments were approved by the Texas A&M Institutional Animal Care and Use Committee.

### Pharmacokinetic and Biodistribution Analyses

Pharmacokinetic and biodistribution experiments were carried out using 8- to 11-week male or female C57BL/6 mice that transgenically express human FcγRs.^38^ Lugol solution (0.1%) was added to drinking water 48 h or 72 h before injection of radiolabeled proteins.

15 μg ch8-18C5 antibody (^125^I-labeled) in 200 μL 0.1% BSA/PBS was injected i.v. in mice. 24 h later, mice were injected i.v. with a 4-fold molar excess of MOG-Seldeg-PS or controls in 200 μL PBS. Whole-body radioactive counts in the mice were obtained using an Atom Lab 100 dose calibrator (Biodex, Shirley, NY, USA). To determine serum radioactivity levels, we retro-orbitally bled mice using 10 μL capillary tubes (Drummond, Broomall, PA, USA) and radioactive counts (c.p.m.) were obtained by gamma counting (Wizard 2480; PerkinElmer). All radioactive counts were expressed as the percentage of the levels obtained from serum samples and whole-body counting immediately before MOG-Seldeg-PS/control delivery.

For biodistribution studies, mice were treated as above, anesthetized, and intracardially perfused with 20–30 mL 10 U/mL heparin in PBS 6 h after MOG-Seldeg-PS or MOG-Seldeg-FcRn delivery, followed by excision of organs or tissues. Blood samples were collected immediately prior to perfusion. Selected organs and tissues were weighed, and radioactivity was counted in a gamma counter (Wizard2480; PerkinElmer) to determine the percentage of injected dose (%ID) per gram and the %ID per organ.^59^

### Analyses of Serum IgG Levels in Mice

Mice were retro-orbitally bled with 44.7 μL heparinized capillary tubes (VWR International, Hermosa Beach, CA, USA). IgG concentrations in 1:30,000 dilutions of serum in PBS were quantitated by ELISA as described previously.^27^

### IHC

To analyze the distribution of ch8-18C5 in liver cells using IHC, we injected (i.v.) 8-week-old male or female mice with 15 μg ch8-18C5. 24 h later, MOG-Seldeg-PS, MOG-Seldeg-FcRn, or controls were i.v. delivered in 200 μL PBS at a 4-fold molar excess. 6 h later, mice were anesthetized and intracardially perfused with 20–30 mL 10 U/mL heparin in PBS, followed by excision of livers. The livers were immediately embedded in Tissue-Tek OCT compound (Sakura Finetek, Torrance, CA, USA), frozen and stored at −80°C. 8 μm sections were prepared and fixed in cold acetone (−20°C) for 2 min. After washing with PBS, sections were incubated in 3% BSA/PBS, followed by incubation with rabbit anti-human IgG Fab’_2_ (LSBio, Seattle, WA, USA) and rat anti-mouse CD31 (clone 390 and/or MEC13.3; BioLegend) or rat anti-mouse F4/80 (clone CI:A3-1; Abcam, Cambridge, MA) diluted in 3% BSA in PBS. Following washes with PBS containing 0.05% Tween 20, the sections were incubated with 1% goat serum (Sigma-Aldrich) for 30 minutes. Bound primary antibodies were detected using cross-adsorbed Alexa 555-labeled polyclonal goat anti-rat IgG (Biolegend) and Alexa 647-labeled polyclonal goat anti-rabbit IgG (Thermo Fisher Scientific) in 5% goat serum in PBS. Following washing with PBS containing 0.05% Tween 20, coverslips were mounted using Vectashield mounting medium containing DAPI (Vector Laboratories, Burlingame, CA, USA).

Sections were imaged using a Zeiss Axio Observer Z1 inverted epifluorescence microscope as described previously.^49^ Images were acquired with filter sets for DAPI (DAPI-5060C-ZHE), Alexa Fluor 555 (Cy3-4040C-ZHE), and Alexa Fluor 647 (Cy5-4040C-ZHE) from Semrock using a Hamamatsu Orca ER CCD camera. The data were processed and displayed using in-house written software, MIATool. To allow comparison between different conditions, we used the same lamp intensity and exposure times for acquiring data for the Alexa 647 channel. Images obtained using secondary antibody only for each individual tissue were used for background level adjustment.

### Treatment of EAE

8- to 11-week-old female mice were immunized subcutaneously at four sites in the flanks with a total of 100 μg/mouse hMOG35-55 peptide (CS Bio, Menlo Park, CA, USA) emulsified with complete Freund’s adjuvant (CFA; Sigma-Aldrich) containing an additional 4 mg/mL heat-inactivated Mycobacterium tuberculosis (strain H37RA, Becton Dickinson, Franklin Lakes, NJ, USA). 200 ng pertussis toxin was administered intraperitoneally at 0 and 48 h post-immunization. The mice were monitored and scored daily for clinical signs of EAE. On day 15, mice were divided into groups based on their clinical signs of EAE (EAE scores) including scores prior to and on day 15, with similar scores per group. Mice were injected i.v. with 250 μg polyclonal IgG from human serum (HC) or 250 μg polyclonal IgG isolated from serum of an MS patient (MS-3) that contains MOG-specific antibodies.^17^ 2 h following MS-3 or HC delivery, mice were i.v. injected with 50 μg MOG-Seldeg-PS, MOG-Seldeg-FcRn, or controls. Mice were monitored and scored daily until day 35. The scoring system for disease activity was as described previously:^60^ 0, no paralysis; 1, limp tail; 2, moderate hind limb weakness; 3, severe hind limb weakness; 4, complete hind limb paralysis; 5, quadriplegia; and 6, death.

### Statistical Analyses

Tests for statistical significance between groups were carried out using unpaired Students t test, one-way ANOVA or two-way ANOVA with Tukey’s multiple comparisons test in GraphPad Prism (GraphPad Software, La Jolla, CA, USA). p values of less than 0.05 were taken to be significant.

## Supplemental Information

Document S1. Figures S1–S5

Document S2. Article plus Supplemental Information
