# Supplementary material for: Selective Depletion of Antigen-Specific Antibodies for the Treatment of Demyelinating Disease
Source: Mol Ther. 2020 Nov 17;29(3):1312–23. doi: 10.1016/j.ymthe.2020.11.017 (PMC7934575; doi:10.1016/j.ymthe.2020.11.017)
Supplement: Document S1. Figures S1–S5 [file mmc1.pdf]

YMTHE, Volume *29*

Supplemental Information

Selective Depletion of Antigen-Specific Antibodies for the Treatment of Demyelinating Disease

Wei Sun, Priyanka Khare, Xiaoli Wang, Dilip K. Challa, Benjamin M. Greenberg, Raimund J. Ober, and E. Sally Ward

Supplemental figures and legends

Supplemental figure 1

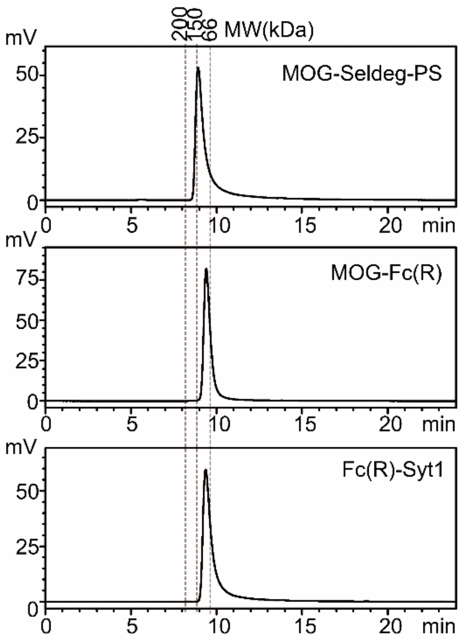

Figure S1. The purified Fc fusions have no detectable aggregates. Gel filtration chromatography analyses of purified Fc fusions with elution times of molecular weight (MW) standards indicated by dotted black lines.

Supplemental figure 2

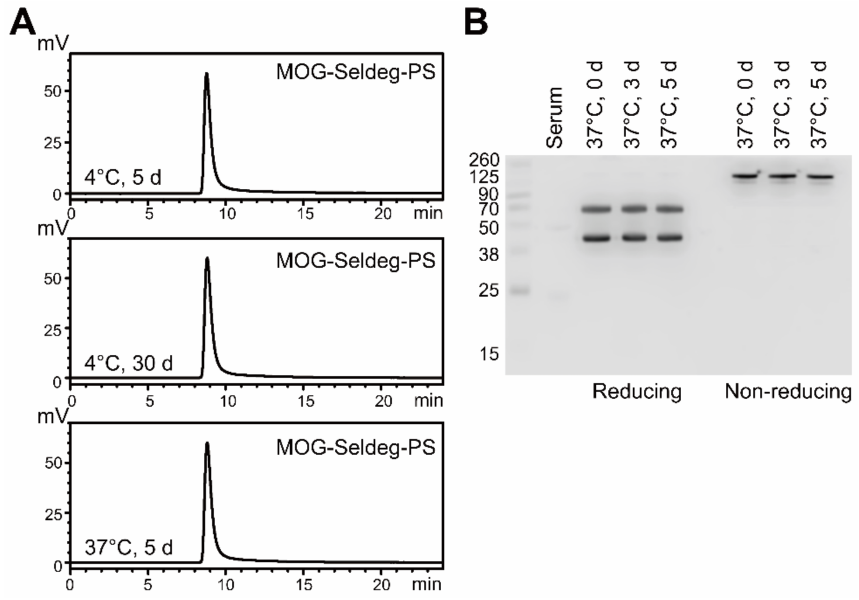

Figure S2. **Stability analyses of MOG-Seldeg-PS**. (**A**) MOG-Seldeg-PS was stored in PBS at 37°C for 5 days (5 d) or 4°C for 5 (5 d) or 30 days (30 d) followed by analyses using a Phenomenex Yarra SEC-3000 column. (**B**) MOG-Seldeg-PS was incubated at 37°C for 0, 3 or 5 days in IgG-depleted human serum at a concentration of 400 nM. Following incubation, MOG-Seldeg-PS was immunoprecipitated using goat anti-human IgG (Fc-specific)-agarose beads and analyzed by immunoblotting using goat anti-human (H+L) antibody conjugated to HRP. Sizes of molecular weight standards are shown in kDa on the left margin. Data shown are representative of two independent experiments.

Supplemental figure 3

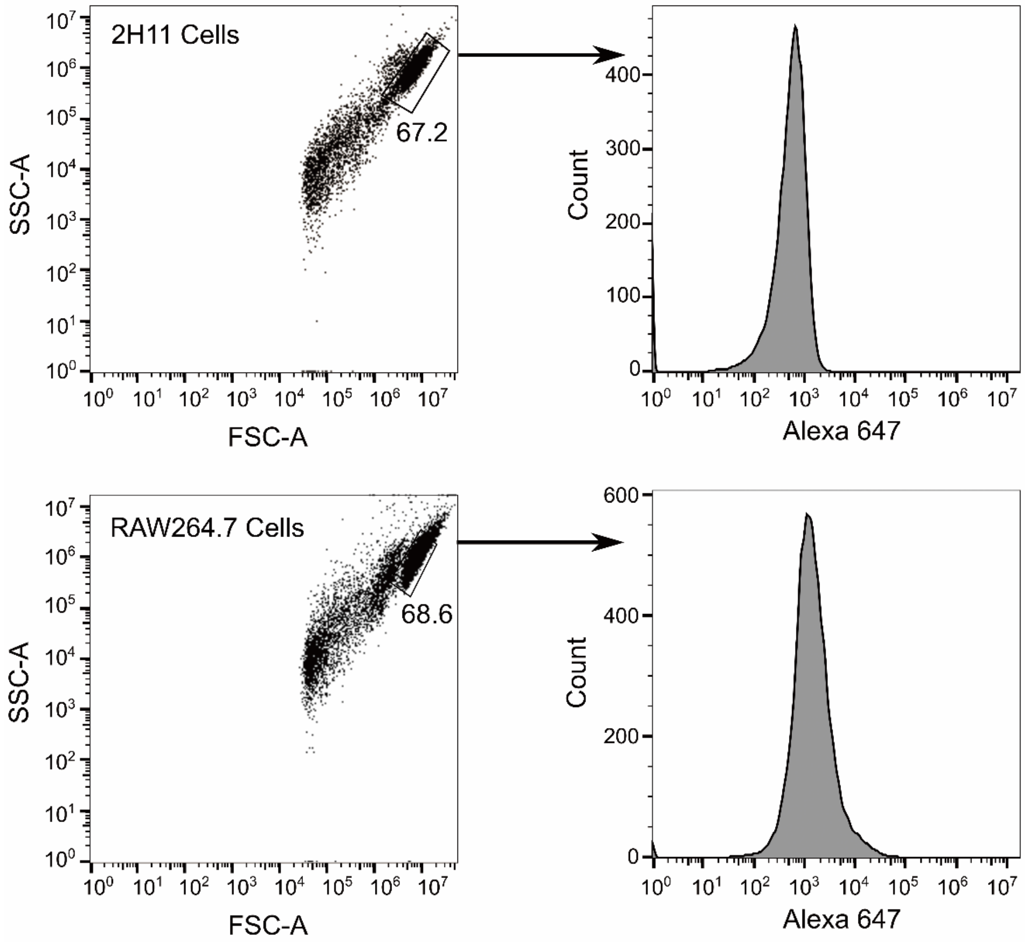

Figure S3. **Gating strategy used in flow cytometry.** The plots show representative gating strategies, using forward scatter area (FSC-A) and side scatter area (SSC-A) displays, to identify live 2H11 and RAW264.7 cell populations for the analyses presented in Figure 1D, E, and Figure 2A.

Supplemental figure 4

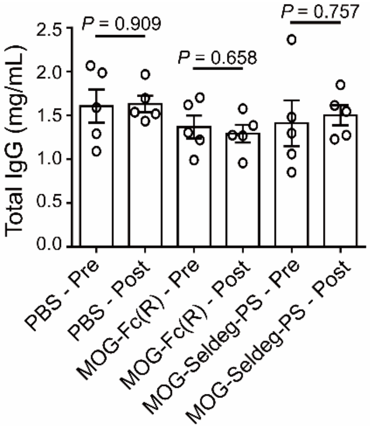

Figure S4. **Delivery of MOG-Seldeg-PS does not affect total serum IgG levels in mice.** Serum samples were isolated from mice immediately prior to ('pre'), and 48 hours following ('post'), injection of MOG-Seldeg-PS (40 μg), MOG-Fc(R) (31 μg) or PBS. Serum IgG levels were determined by ELISA and mean values for each time point/mouse group (n = 5 mice/group) are shown. Error bars indicate SEM. Differences for IgG levels before and after treatment were not significant ($P > 0.05$; unpaired two-tailed Student's t-test). Data shown are representative of two independent experiments.

Supplemental figure 5

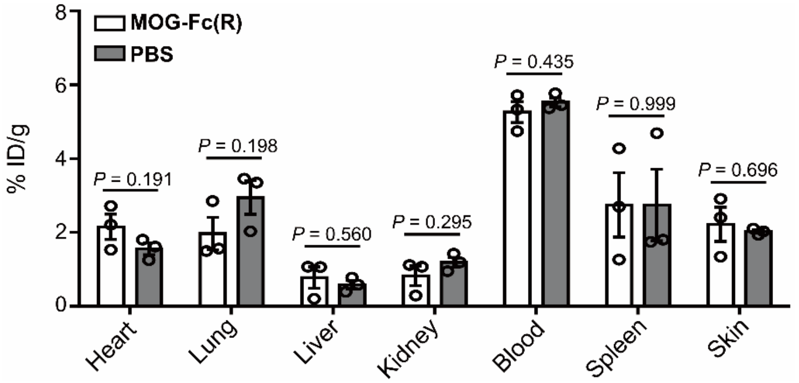

Figure S5. **Treatment with control protein, MOG-Fc(R) or PBS vehicle has similar effects on the biodistribution of MOG-specific antibody.** Transgenic mice expressing huFcγRs were intravenously injected with radiolabeled (125-I) ch8-18C5 (15 μg). 24 h following ch8-18C5 delivery, mice were intravenously injected with MOG-Fc(R) (31 μg) or PBS vehicle (n = 3 mice/group). Blood and organs were harvested 6 h following MOG-Fc(R) or PBS delivery (30 h after injection of ch8-18C5). Data shown are mean values of percentage injected dose per gram (% ID/g) for blood or organs. Error bars indicate SEM and *P* values indicating no significant differences ($P > 0.05$; unpaired two-tailed Student's t-test) between the two treatments are shown.
